# Supplementary material for: Ehrlichia chaffeensis Etf-3 Induces Host RAB15 Upregulation for Bacterial Intracellular Growth
Source: Int J Mol Sci. 2024 Feb 22;25(5):2551. doi: 10.3390/ijms25052551 (PMC10931967; doi:10.3390/ijms25052551)
Supplement: Supplementary file 1 [file ijms-25-02551-s001.zip › ijms-2883249-supplementary.pdf]

Supplementary Figures

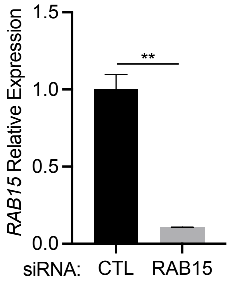

Figure S1. The silence efficiency of RAB15 was determined with qRT-PCR. THP-1 cells were transfected with siRNA targeting *RAB15* or control siRNA for 24 h. The *RAB15* mRNA levels were determined with qRT-PCR and normalized against those of human GAPDH. Relative values to the amount of *RAB15* in control cells are shown. Data indicate means ± standard deviations (n = 3). The significant difference is represented by p-values determined with Student's t-test (** indicates $p < 0.01$).

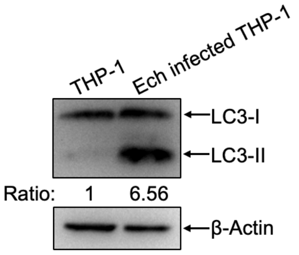

Figure S2. *Ehrlichia chaffeensis* infection induces autophagy in THP-1 cells. THP-1 cells were infected with isolated *E. chaffeensis*. At 48 h p.i., the expression of LC3 was determined using Western blotting. The numbers below the panels indicate the relative intensity of each protein band. The protein level of LC3-II in control cells is set as 1.

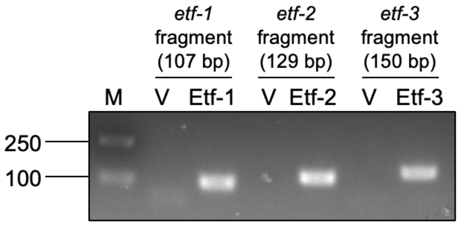

Figure S3. Overexpression of *E. chaffeensis* T4SS effectors in THP-1 cells. THP-1 cells were transfected with pcDNA3.1 overexpressing Etf-1, Etf-2 or Etf-3 for 48 h. Total RNA was prepared from the transfected cells. The expression of T4SS effector was determined with RT-PCR. Names and sizes of amplified products are indicated above. M, molecular size marker. V, THP-1 cells transfected with pcDNA3.1 vector. Etf-1, Etf-2 or Etf-3, THP-1 cells transfected with pcDNA3.1 overexpressing Etf-1, Etf-2 or Etf-3, respectively.

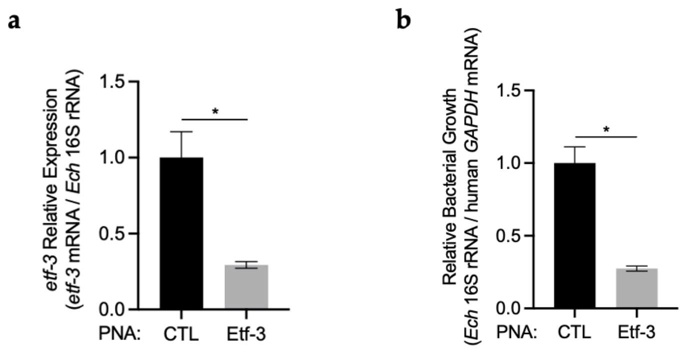

Figure S4. Transfection of Etf-3 PNA significantly reduces *E. chaffeensis etf-3* mRNA level and bacterial intracellular growth. THP-1 cells were synchronously infected with Etf-3 PNA-transfected *E. chaffeensis*. At 48 h p.i., total RNA was prepared from the infected cells. (**a**) The levels of *etf-3* mRNA were determined with qRT-PCR and normalized against those of *E. chaffeensis* 16S rRNA. Relative values to the amount of CTL PNA transfected *E. chaffeensis* are shown. (**b**) Relative bacterial numbers were determined as the levels of bacterial 16S rRNA normalized against those of human *GAPDH* mRNA. Relative values to the amount of CTL PNA transfected *E. chaffeensis* are shown. Data indicate means ± standard deviations (n = 3). The significant differences are represented by p-values determined with Student's t-test (* indicates $p < 0.05$).

Supplementary Tables

Table S1. Bacterial strains and plasmids used in this study.

Strains/Plasmids	Description	Source (Reference)
***E. coli* strains**		
DH5α/pcDNA3.1	DH5α harbouring pcDNA3.1 plasmid; Amp^r	This study
DH5α/pcDNA3.1-Etf-1-FLAG	DH5α harbouring pcDNA3.1-Etf-1-FLAG plasmid; Amp^r	This study
DH5α/pcDNA3.1-Etf-2-FLAG	DH5α harbouring pcDNA3.1-Etf-2-FLAG plasmid; Amp^r	This study
DH5α/pcDNA3.1-Etf-3-FLAG	DH5α harbouring pcDNA3.1-Etf-3-FLAG plasmid; Amp^r	This study
**Plasmids**		
pcDNA3.1	Clone vector; Amp^r	Invitrogen
pcDNA3.1-Etf-1-FLAG	pcDNA3.1 harbouring *etf-1* gene; Amp^r	This study
pcDNA3.1-Etf-2-FLAG	pcDNA3.1 harbouring *etf-2* gene; Amp^r	This study
pcDNA3.1-Etf-3-FLAG	pcDNA3.1 harbouring *etf-3* gene; Amp^r	This study

Amp^r, ampicillin resistance.

Table S2. Primers used in this study.

Primers	Sequence (5'→3')	Function
GAPDH-qRT-F	TCAAGGCTGAGAACGGGAAG	qRT-PCR
GAPDH-qRT-R	TGGACTCCACGACGTACTCA	qRT-PCR
16S rRNA-qRT-F (*E. chaffeensis*)	GGTGAGTAATGCGTAGGAATC	qRT-PCR
16S rRNA-qRT-R (*E. chaffeensis*)	GCTCATCTAATAGCGATAAATC	qRT-PCR
RAB15-qRT-F	GCTGTTCCGGCTGCTGCTGAT	qRT-PCR
RAB15-qRT-R	CCCGCCGATAGTACTGCTTTGTGA	qRT-PCR
etf-1-qRT-F	GCGTAGGTAAATACCTTGGAGGCA	qRT-PCR and RT-PCR
etf-1-qRT-R	CAGTTTGTGGGTACGGTTGTGCAT	qRT-PCR and RT-PCR
etf-2-qRT-F	CAGCAACAAGTACCGCAAAC	qRT-PCR and RT-PCR
etf-2-qRT-R	AGGTGGTCTCCTGTTTGTTATC	qRT-PCR and RT-PCR
etf-3-qRT-F	GCCCCTAATTGATGATAGAACCTC	qRT-PCR and RT-PCR
etf-3-qRT-R	TGCGTGTTGTGAGCAGAGTTA	qRT-PCR and RT-PCR
Etf-1-FLAG-F	CGGGATCCATGCTTACTTTCTTAAAGAAAGGTG	construct vector
Etf-1-FLAG-R	GGAATTCTTATTTATCATCATCATCTTTGTAATCTCTTGCAT GTACTTTTCCTGG	construct vector
Etf-2-FLAG-F	CGGGATCCATGCCAACAACACTAGGGACTG	construct vector
Etf-2-FLAG-R	GGAATTCTTATTTATCATCATCATCTTTGTAATCTCTGCGAA AAAATCTACCACAT	construct vector
Etf-3-FLAG-F	CGGGATCCATGTGTAATAATGCTATGCTAAC	construct vector

Etf-3-FLAG-R	GGAATTCTTATTTATCATCATCATCTTTGTAATCTCTTGCTATTGTTCTTGTAA	construct vector
